# Influence of epilepsy and antiepileptic drug intake in patients suffering from aneurysmal subarachnoid hemorrhage on outcome

**DOI:** 10.1016/j.bas.2025.105924

**Published:** 2025-12-29

**Authors:** Tim Lampmann, Harun Asoglu, Haitham Alenezi, Mohammed Jaber, Bettina Otto, Mohammed Banat, Erdem Güresir, Hartmut Vatter, Motaz Hamed

**Affiliations:** a Department of Neurosurgery, University Hospital Bonn, Venusberg Campus 1, Bonn, 53127, Germany; b Neurological Rehabilitation Center Godeshöhe, Waldstraße 2-10, Bonn, 53177, Germany

**Keywords:** SAH, Seizure, AED

## Abstract

**Objective:**

Many patients suffering from aneurysmal subarachnoid hemorrhage (SAH) develop epileptic seizures. The recent guidelines do not recommend routine administration of antiepileptic drugs (AED).

**Research question:**

We performed a retrospective single-center study to analyze the effect of AEDs on the outcome in patients suffering from epilepsy after SAH.

**Methods:**

752 patients with SAH treated between 01/2006 and 12/2020 were analyzed. Patients were divided into good-grade (WFNS grades I-II) versus poor-grade (WFNS grades III-V) on admission. Data of patients’ history as well as clinical course were collected. Outcome according to the modified Rankin scale (mRS) score was assessed at 6 months after ictus. Outcome was dichotomized into favorable (mRS 0–2) and unfavorable (mRS 3–6). Univariate and multivariate analyses were performed.

**Results:**

346 (46.0 %) patients suffered from poor-grade SAH and 366 (48.7 %) patients achieved unfavorable outcome. 202 (26.9 %) patients suffered from seizures after SAH and 136 (18.1 %) had to be treated with antiepileptic drugs (AEDs) for more than a week. Epilepsy and AED intake after 3 months was more often in patients with unfavorable outcome (18.9 % vs. 8.3 %; p < 0.001 and 21.9 % vs. 11.9 %; p < 0.001, respectively).

In multivariate analysis, ‘poor-grade SAH’ (p < 0.001, OR 10.5, 95 % CI 6.0–18.2), ‘age >50 years’ (p = 0.001, OR 2.7, 95 % CI 1.5–4.8, ‘aneurysm size >10 mm’ (p = 0.018, OR 2.2, 95 % CI 1.1–4.1), ‘hydrocephalus’ (p = 0.002, OR 2.6, 95 % CI 1.4–4.7), ‘delayed cerebral ischemia’ (p = 0.002, OR 5.0, 95 % CI 2.3–10.9) and ‘epilepsy within 3 months’ (p = 0.002, OR 5.9, 95 % CI 1.9–18.3) were predictors for unfavorable outcome, whereas ‘AED intake after 6 months’ (p = 0.037, OR 0.35, 95 % CI 0.13–0.94) was predictor for favorable outcome.

**Conclusions:**

Manifestation of epilepsy in patients suffering from SAH deteriorates outcome. Continued AED intake in SAH patients who developed epileptic seizures should be advised.

## Highlights

• Post-SAH epilepsy occurs in about 14 % of patients.
• Epilepsy in patients suffering from SAH is associated with unfavorable outcome.
• Continued AED intake should be advised in SAH patients who suffer from epilepsy.

## 1 Introduction

Morbidity and mortality are still high in patients suffering from aneurysmal subarachnoid hemorrhage (SAH) (Neifert et al., 2021). Despite an increased survival in the past few decades, SAH patients may suffer from permanent impairment mostly driven by delayed cerebral ischemia (DCI) (Neifert et al., 2021; Macdonald and Schweizer, 2017). The most important SAH treatment is early aneurysm repair and the only pharmacological therapy for preventing DCI is nimodipine (Neifert et al., 2021; Hoh et al., 2023; Allen et al., 1983). Nevertheless, SAH patients are at high risk of encountering many possible complications like hydrocephalus, cerebral vasospasms and medical complications (Neifert et al., 2021). Beside these, onset seizures are also a risk factor for unfavorable outcome as well (Butzkueven et al., 2000). For patients who present with seizures at SAH onset, treatment with antiepileptic drugs (AEDs) is recommended for 7 days, but not further (Hoh et al., 2023). For patients who do not suffer from seizures, prophylactic AEDs are generally not recommended (Hoh et al., 2023; Marigold et al., 2013). Currently, optimal management of AEDs in SAH patients is still unclear. The aim of this retrospective single-center study was to evaluate the effect of AEDs on the outcome in patients suffering from epilepsy within SAH.

## 2 Materials and methods

### 2.1 Study design and patient characteristics

**Fig. 1:**
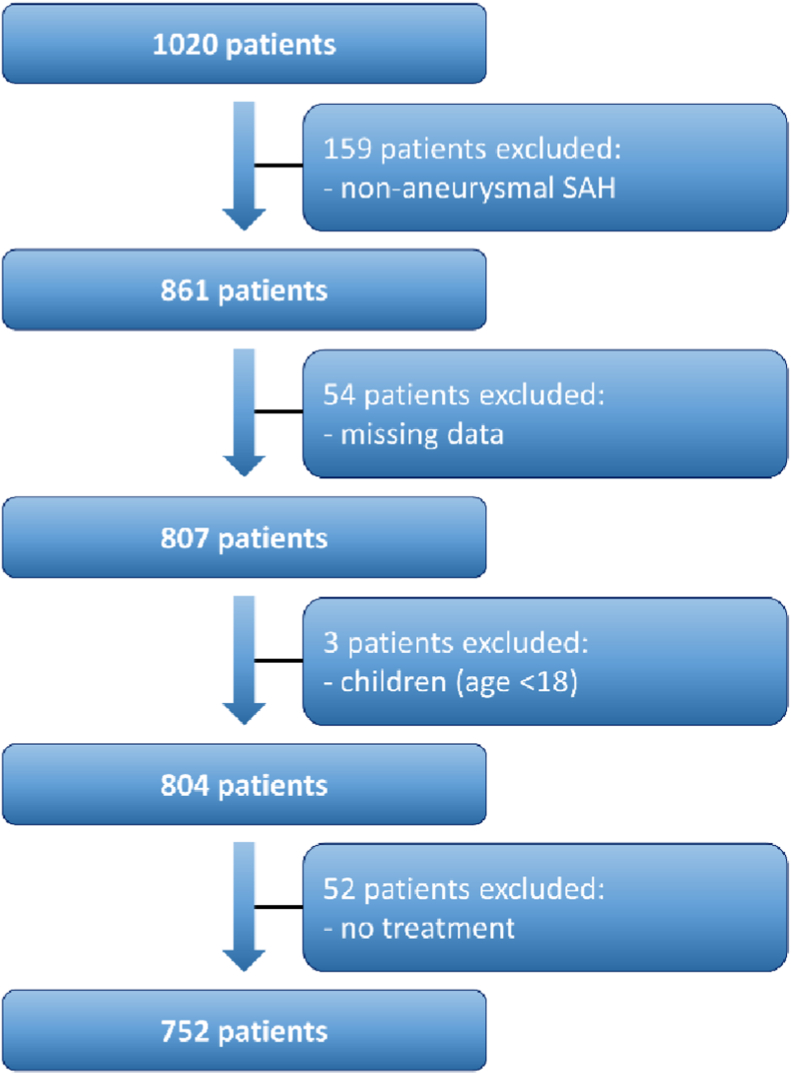
Flow chart illustrating the selection process of consecutive SAH patients.

752 consecutive patients suffering from SAH were treated at the authors’ institution between 01/2006 and 12/2020. Children (<18 years), patients with non-aneurysmal SAH and patients that received no further treatment were excluded. Patient characteristics, medical history, treatment modality, aneurysm size and location were retrospectively assessed. Moreover, information on occurrence of epilepsy and AED intake were gathered for 6 months after SAH. Epilepsy was defined as at least two unprovoked seizures occurring at intervals of >24 h or one unprovoked seizure with an increased probability of further seizures similar to the general risk after two unprovoked seizures according to the International League Against Epilepsy. Data were entered into a computerized database (SPSS, Version 27, IBM Corp., Armonk, NY). Patients for whom detailed data were missing were excluded as well (Fig. 1).

Patients were graded on admission regarding their neurological condition according to the World Federation of Neurosurgical Societies (WFNS) score. Patients were stratified into good-grade (WFNS I–II) versus poor-grade (WFNS III–V). Outcome was measured 6 months after SAH with the modified Rankin Scale (mRS) and dichotomized into favorable (mRS 0–2) versus unfavorable (mRS 3–6). AED intake and manifested seizure disorder were assessed 3 and 6 months after SAH if available.

### 2.2 Clinical management

Patients suffering from thunderclap headache or loss of consciousness underwent computed tomography (CT) or lumbar puncture was performed for diagnosis of SAH. Source of bleeding was verified by CT-angiography or digital subtraction angiography. Optimal treatment modality was interdisciplinary chosen based on patient-level regarding aneurysm location, size and individual patient characteristics like condition, age or additive intracerebral hemorrhage. Aneurysms were treated as soon as possible after admission regardless of WFNS grades. All patients were monitored on the neurosurgical intensive care unit and received medical treatment with nimodipine. In cases of delayed cerebral ischemia or delayed ischemic neurological deficit, hypertension was induced. Seizures at SAH onset were gathered from reports of the emergency physicians. Onset seizures were treated by AEDs for not more than a week, except epilepsy was diagnosed in further course. If epilepsy aka late onset seizures during treatment course was diagnosed by clinical symptoms or electroencephalography (EEG), antiepileptic treatment was initiated. Consultant neurologists decided on antiepileptic treatment. Detailed clinical management of patients suffering SAH at the authors’ institution was reported previously (Lampmann et al., 2022; Vychopen et al., 2023).

### 2.3 Statistical analysis

Data were analyzed using SPSS Statistics (Version 27, IBM Corp. Armonk, NY, USA). Continuous data were expressed as mean values ± standard deviation and categorical variables were expressed as absolute and relative frequencies. Unpaired *t*-test was used to test for differences of continuous data. Categorical variables were analyzed in contingency tables using the exact Fisher's-test. Receiver-operating characteristic (ROC) analysis was performed for selection of best cut-off for continuous data using the Youden index before dichotomization. For multivariate analysis a backward stepwise method was used to construct a multivariable logistic regression model in relation of an unfavorable outcome (mRS 3–6) as an dependent variable with an inclusion criterion of p-value <0.05.

## 3 Results

**Fig. 2:**
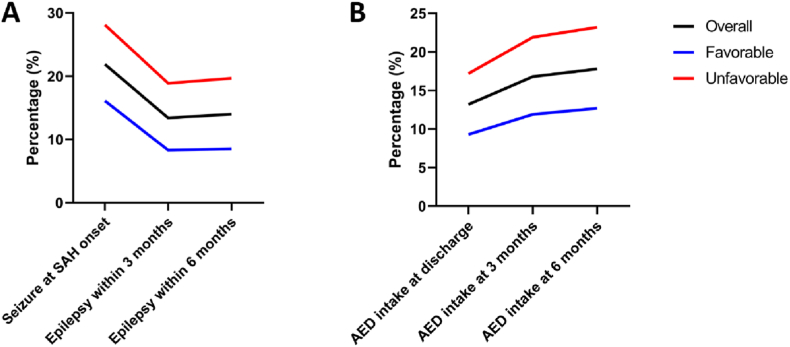
Line chart representing development of epilepsy (**A**) and AED intake (**B**) over time.

346 (46.0 %) patients suffered from poor-grade SAH and 366 (48.7 %) patients achieved unfavorable outcome. 165 (21.9 %) patients presented with initial seizure at SAH onset. None of the patients had AEDs in their premedication. 136 (18.1 %) patients received AEDs and all of them suffered from at least one seizure attack. 99 (13.2 %) patients were discharged with AED, but 126 (16.8 %) patients had to take AED 3 months after SAH. Epilepsy and AED intake after 3 months was more often in patients with unfavorable outcome (18.9 % vs. 8.3 %; p < 0.001 and 21.9 % vs. 11.9 %; p < 0.001, respectively). Baseline characteristics and results of the univariate analyses are shown in Table 1. Fig. 2 visualizes the AED intake and development of epilepsy in time course.

**Table 1:** Patient characteristics according to their outcome.

	Overall	Favorable Outcome (mRS 0–2)	Unfavorable Outcome (mRS 3–6)	p-value
**Overall**	752 (100 %)	386 (51 %)	366 (49 %)	

**Mean Age (± SD) [in years]**	55.6 ± 13	52.5 ± 12	58.9 ± 13	**<0.001**

**Sex**				
Female	501 (67 %)	257 (67 %)	244 (67 %)	1.000
Male	251 (33 %)	129 (33 %)	122 (33 %)	

**Poor-grade SAH (WFNS grade III-V)**	346 (46 %)	79 (21 %)	267 (73 %)	**<0.001**

**Aneurysm location**				0.094
ACOM/ACA	308 (41 %)	166 (43 %)	142 (39 %)	
ICA	168 (22 %)	90 (23 %)	78 (21 %)	
MCA	167 (22 %)	86 (22 %)	81 (22 %)	
Posterior Circulation	109 (15 %)	44 (12 %)	65 (18 %)	

**Mean aneurysm size (± SD) [in mm]**	7.7 ± 5.1	6.8 ± 3.4	8.8 ± 6.2	**<0.001**

**Treatment**				0.132
Surgical	340 (45 %)	166 (43 %)	174 (47 %)	
Endovascular	410 (55 %)	220 (57 %)	190 (52 %)	
Combined	2 (0 %)	0 (0 %)	2 (1 %)	

**Seizure at SAH onset**	165 (22 %)	62 (16 %)	103 (28 %)	**<0.001**

**AED intake at discharge**	99 (13 %)	36 (9 %)	63 (17 %)	**0.002**

**Epilepsy within 3 months**	101 (12 %)	32 (8 %)	69 (19 %)	**<0.001**

**AED intake at 3 months**	126 (17 %)	46 (12 %)	80 (22 %)	**<0.001**

**Epilepsy within 6 months**	105 (14 %)	33 (9 %)	72 (20 %)	**<0.001**

**AED intake at 6 months**	134 (18 %)	49 (13 %)	85 (23 %)	**<0.001**

ACA: anterior cerebral artery; ACOM: anterior communicating artery; AED: antiepileptic drugs; ICA: internal carotid artery; MCA: middle cerebral artery; mRS: modified Rankin scale; SD: standard deviation; WFNS: World Federation of Neurosurgical Societies. Significant p-values <0.05 are marked bold.

**Fig. 3:**
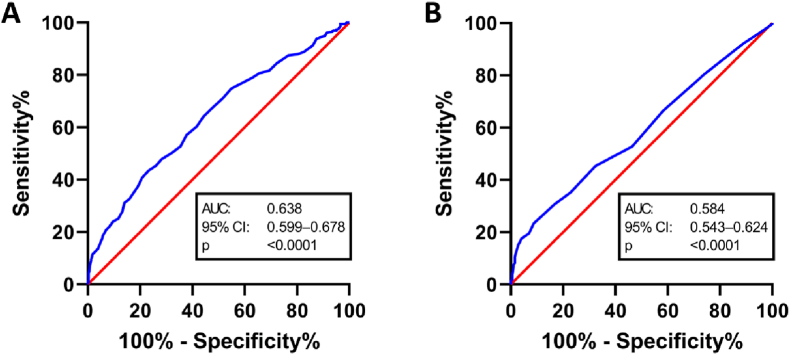
Receiver-operating characteristic curve illustrating age (**A**) and aneurysms size (**B**) in prediction of unfavorable outcome.

**Fig. 4:**
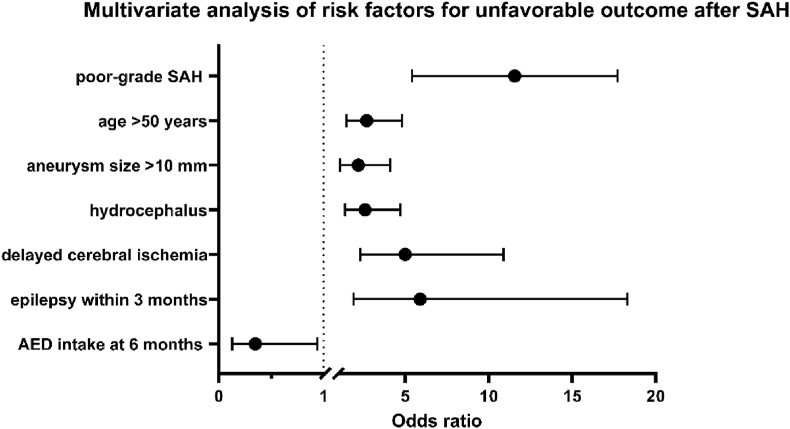
Forest plot presenting the result of multivariable logistic regression analysis for an unfavorable outcome (modified Rankin scale 3–6) within the study group of patients suffering from SAH. AED: antiepileptic drugs; SAH: subarachnoid hemorrhage.

A multivariable logistic regression analysis was performed to predict unfavorable outcome (modified Rankin scale 3–6) after SAH. ROC analysis was performed to select the optimal cut-off for continuous data (Fig. 3). The following potentially independent variables were included in the analysis: Age (≤50/>50 years), aneurysm size (≤10/>10 mm), WFNS grade (good/poor), hydrocephalus, cerebral vasospasms, delayed cerebral ischemia, AED intake at discharge, 3 and 6 months after SAH as well as seizures at SAH onset and epilepsy 3 and 6 months after SAH. According to multivariate analysis ‘poor-grade SAH’ (p < 0.001, OR 10.5, 95 % CI 6.0–18.2), ‘age >50 years’ (p = 0.001, OR 2.7, 95 % CI 1.5–4.8, ‘aneurysm size >10 mm’ (p = 0.018, OR 2.2, 95 % CI 1.1–4.1), ‘hydrocephalus’ (p = 0.002, OR 2.6, 95 % CI 1.4–4.7), ‘delayed cerebral ischemia’ (p = 0.002, OR 5.0, 95 % CI 2.3–10.9) and ‘epilepsy within 3 months’ (p = 0.002, OR 5.9, 95 % CI 1.9–18.3) were predictors for unfavorable outcome, whereas ‘AED intake after 6 months’ (p = 0.037, OR 0.35, 95 % CI 0.13–0.94) was predictor for favorable outcome (Nagelkerke's R^2^ 51.2 %). The significant results were visualized in Fig. 4.

## 4 Discussion

We present a retrospective single-center study that analyzes the effect of AEDs on the outcome in patients who developed epilepsy after SAH. 752 patients could be included in this analysis. Patients were treated for SAH following the currently available institutional and international guidelines (Hoh et al., 2023; Connolly et al., 2012). When SAH patients suffered from seizures or EEG revealed convulsive status epilepticus or non-convulsive status epilepticus, AEDs were given according to national guidelines. We evaluated AED intake and development of epilepsy in the follow-up. Beside commonly known risk factors for an unfavorable outcome like age or poor-grade SAH, AED intake and seizure occurrence were significantly associated with the outcome (Catapano et al., 2021; Report of World Federation of, 1988). Patients who developed epilepsy within 3 months after SAH had a significantly worse outcome, whereas AED intake may be a protective factor against unfavorable outcome. In the study cohort, more patients took AEDs 3 months after SAH as compared to discharge (16.8 % vs. 13.2 %).

The overall (from onset to follow-up at 6 months) seizure incidence of 26.9 % was comparable to other studies (Chen et al., 2021; Huttunen et al., 2015; Rhoney et al., 2000; O'Connor et al., 2014). Known risk factors for seizure are MCA aneurysms, high clinical or radiological grade, cortical infarction or hydrocephalus (Hoh et al., 2023). Patients with an unfavorable outcome suffered significantly more often from seizures at SAH onset (28.1 % vs. 16.1 %; p < 0.001) that is described in other studies as well (Butzkueven et al., 2000; Claassen et al., 2003). Notably, this effect vanished in multivariate analysis. According to the multivariate analysis, development of epilepsy, with the onset of seizures occurring either at the time of SAH or within the first 3 months, was independently associated with unfavorable outcome. On the other hand, our data suggest an improved outcome when SAH patients are on AEDs even after 6 months (odds ratio for unfavorable outcome: 0.35, 95 % CI 0.13–0.94; p = 0.037). However, all of the patients that received AEDs suffered from at least one seizure attack. So our data emphasizes that patients who suffer from SAH-related seizures may benefit from AED prescription. In contrast to our findings, Chen et al. found that only a short-term AED intake (no more than 3 days) resulted in a better outcome than long-term use, but the authors investigated a prophylactic approach (Chen et al., 2021).

In fact, most studies focus on prophylaxis, but routine use of prophylactic AEDs in SAH patients in order to improve patients' outcome is still inconclusive, mostly because of a lack of randomized controlled trials (Hoh et al., 2023; Marigold et al., 2013). Some studies investigated the effect of long-term use of prophylactic AED intake that seems to be associated with an unfavorable outcome whereas seizure risk was not decreased (Chen et al., 2021; Panczykowski et al., 2016). Given this risk/benefit ratio, routine use of prophylactic AEDs in SAH patients is not recommended in the current guideline (Hoh et al., 2023). In the past, phenytoin or valproic acid were administered commonly, but these may harm the patients because of side effects (Raper et al., 2013). Since morbidity seems to be higher in phenytoin compared to other AEDs, the use of phenytoin is no longer recommended in SAH patients (Hoh et al., 2023; Naidech et al., 2005). According to this, levetiracetam is mostly used as first line AED at the authors’ institution. Due to the retrospective design of the present study, detailed information of which AEDs were used were not available in many cases so it was omitted. The use of phenytoin may have biased previous studies that advised against a prophylactic antiepileptic therapy. Therefore, Chen et al. performed a meta-analysis of AED prophylaxis in SAH patients, but could not prove superiority of levetiracetam in comparison to phenytoin because of the limited available data (Chen et al., 2021). In contrast, a prospective study including TBI and SAH showed an improved outcome for patients who received levetiracetam instead of phenytoin (Szaflarski et al., 2010). We assume that not specifying individual AEDs is an advantage of the present study: Independently of the used AED, patients with SAH-related epilepsy who continued AED intake may achieve more favorable outcome. Although it seems obvious to treat epilepsy by AED, this study showed a positive influence on outcome as well.

Many studies just analyzed epilepsy during the hospital stay. Choi et al. already described that seizures at SAH onset were not predictive for late-onset epilepsy (Choi et al., 2009). In our study cohort 7 patients did neither present with seizure at SAH onset nor after 3 months, but developed epilepsy within 6 months after SAH. 57 % of them achieved unfavorable outcome. In a Finish follow-up study cumulative incidence of epilepsy increased from 8 % at 1 year to 12 % at 5 years (Huttunen et al., 2015). This is comparable to ischemic stroke and intracerebral hemorrhage as well (Galovic et al., 2018; Haapaniemi et al., 2014). This emphasizes the importance of a long-term evaluation and the necessary awareness for late-onset epilepsy. On the other hand, epilepsy within 6 months after SAH was no independent predictor for unfavorable outcome like epilepsy within 3 months was.

While the current international SAH guideline only advise for treatment of early-onset seizures, the present study focused on antiepileptic therapy for patients suffering from SAH-related epilepsy (Hoh et al., 2023). Choi et al. already described that onset seizures are a distinct entity from late-onset epilepsy (Choi et al., 2009). The present study affirms treatment of epilepsy of SAH patients by AED.

### 4.1 Limitations

The retrospective and single-center design of this study is clearly a limitation. Information of older data were often missing due to lacking (electronic) documentation in the past. Moreover, information of prescribed AEDs were also missing in many cases so it could not be analyzed in this study. Information of indications for AED intake and the reasons for stopping or continuing AED intake were also not available.

## 5 Conclusions

Epilepsy in patients suffering from SAH is associated with unfavorable outcome. Continued AED intake should be advised in SAH patients who suffer from epilepsy. It remains unclear why AED prescription was aborted in some cases, albeit this study assumes a potentially advantage of continued AED intake.

## Ethical approval

This study was conducted according to the guidelines of the Declaration of Helsinki and approved by the Ethics Committee of the Medical Faculty and the University Hospital of Bonn (code: 2024-460-BO). Informed consent was waived due to the retrospective study design.

## Clinical trial number

Not applicable.

## Author contributions

MH conceived the study. TL and MH collected the data. TL and MH analyzed the results. TL made the visualizations. TL and HAs drafted the manuscript. HAl, MJ, BO, MB, EG, HV and MH reviewed and edited the manuscript. All authors have read and agreed to the published version of the manuscript.

## Data availability statement

All data generated or analyzed during this study are included in this published article.

## Funding

No funding was received to assist with the preparation of this manuscript.

## Declaration of competing interest

The authors declare that they have no known competing financial interests or personal relationships that could have appeared to influence the work reported in this paper.
